# Strong, Tough, and Adhesive Polyampholyte/Natural Fiber Composite Hydrogels

**DOI:** 10.3390/polym14224984

**Published:** 2022-11-17

**Authors:** Yongqi Yan, Longya Xiao, Qin Teng, Yuanyuan Jiang, Qin Deng, Xuefeng Li, Yiwan Huang

**Affiliations:** 1 Hubei Provincial Key Laboratory of Green Materials for Light Industry, Hubei University of Technology, Wuhan 430068, China; 2 New Materials and Green Manufacturing Talent Introduction and Innovation Demonstration Base, Hubei University of Technology, Wuhan 430068, China; 3 Non-Power Nuclear Technology Collaborative Innovation Center, Hubei University of Science and Technology, Xianning 437100, China; 4 Hubei Longzhong Laboratory, Xiangyang 441000, China

**Keywords:** polyampholyte gel, cellulose-based fiber, strengthening, toughening, adhesion

## Abstract

Hydrogels with high mechanical strength, good crack resistance, and good adhesion are highly desirable in various areas, such as soft electronics and wound dressing. Yet, these properties are usually mutually exclusive, so achieving such hydrogels is difficult. Herein, we fabricate a series of strong, tough, and adhesive composite hydrogels from polyampholyte (PA) gel reinforced by nonwoven cellulose-based fiber fabric (CF) via a simple composite strategy. In this strategy, CF could form a good interface with the relatively tough PA gel matrix, providing high load-bearing capability and good crack resistance for the composite gels. The relatively soft, sticky PA gel matrix could also provide a large effective contact area to achieve good adhesion. The effect of CF content on the mechanical and adhesion properties of composite gels is systematically studied. The optimized composite gel possesses 35.2 MPa of Young’s modulus, 4.3 MPa of tensile strength, 8.1 kJ m^−2^ of tearing energy, 943 kPa of self-adhesive strength, and 1.4 kJ m^−2^ of self-adhesive energy, which is 22.1, 2.3, 1.8, 6.0, and 4.2 times those of the gel matrix, respectively. The samples could also form good adhesion to diverse substrates. This work opens a simple route for fabricating strong, tough, and adhesive hydrogels.

## 1. Introduction

Polymeric hydrogel is a three-dimensional hydrophilic network containing a lot of water [1,2]. The soft and wet nature of hydrogels endows them with great application potential in diverse areas such as flexible electronics, soft robotics, biomedical devices, wound dressing, and tissue engineering [3,4,5,6,7,8]. Most of these applications generally require hydrogels to possess good mechanical properties to bear relatively high loads and to resist cracks effectively. Yet, conventional hydrogels are usually mechanically weak and lack crack resistance [9]. Recent research has made significant progress in the design and fabrication of strong and tough hydrogels [9,10,11,12,13,14,15,16,17,18,19,20,21,22,23]. Among them, double-network (DN) hydrogels with a tightly crosslinked first network and a loosely crosslinked second network, which could dissipate energy efficiently during deformation, exhibit high strength and toughness [10,11,24,25,26]. Alternatively, the composite strategy is also an effective way to improve the mechanical performance of hydrogels [27,28], where hydrogels act as the matrix and nanomaterials serve as the reinforcement filler. Recently, fiber-reinforced soft composites (FRSCs) have been developed from polyampholyte hydrogels reinforced by woven glass fabrics [29,30,31]. Although these FRSCs exhibit excellent strength and toughness, they have limited fracture strain (within 10%), limiting their practical applications.

In addition, strong adhesion of hydrogels—either among themselves or to soft and hard substrates—is also required for many of these applications. For example, a hydrogel film should adhere stably on soft skins when used as a wound dressing. Another example is that hydrogels should be integrated with soft or hard elements by good adhesions when they are applied especially for flexible electronics and soft robotics. However, a thin water-molecular layer usually exists on the surface of traditional hydrogels (including DN gels) because of their high-water content, making them have high lubrication and a low adhesion with most the substrates [32,33]. Recently, a chemical anchoring strategy has been developed to achieve tough bonding of hydrogels to non-porous solid surfaces, resulting in high interfacial toughness [34,35]. However, irreversible adhesion after debonding still hinders the applications of such strategy. Polyampholyte (PA) hydrogels are also synthesized from cationic and anionic monomers via radical solution copolymerization, which demonstrates relatively high load-bearing properties and good crack resistance (tearing energy ≈ 3000 J m^−2^) due to dynamic ionic bonds [12]. The self-adjustable ionic network of PA gels endows them with reversible adhesion with diverse surfaces as well. Yet, PA gels alone still possess relatively low mechanical properties (e.g., low modulus).

In fact, conflicts exist between mechanical properties and adhesion in a material. According to an experiential adhesion theory [36], the following scaling relationship should be followed for the design of an adhesive with high capacity: $F_{c}\;\sim \;\sqrt{G_{c}}\;\sqrt{A/C}$, where *G*_c_ is the critical strain energy release rate for the interface, *A* is the real area of contact between the adhesive and the substrate, and *C* is the compliance (i.e., reciprocal of the stiffness) in the direction of loading. Based on this scaling law, effective adhesion needs soft and ductile hydrogels to provide sufficient real contact with solid substrates while these gels are usually mechanically weak. In contrast, relatively strong and stiff hydrogels can only provide limited real contact with the substrates, giving relatively low adhesion. Therefore, achieving high mechanical strength and modulus, high fracture resistance, and good adhesion simultaneously in a hydrogel is very difficult and rarely realized until now.

**Figure 1:**
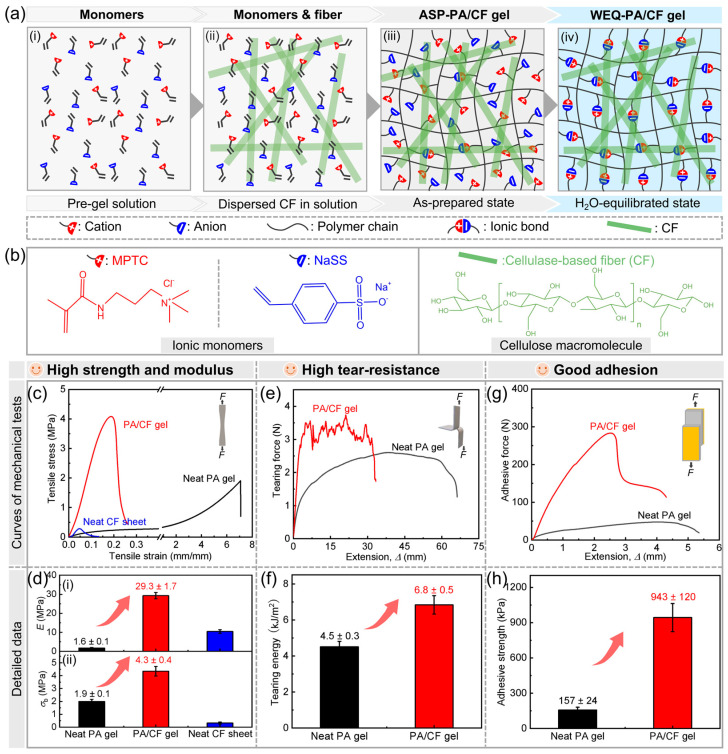
Design, fabrication, and proof of strong, tough, and adhesive PA/CF composite hydrogels. (**a**) Design of the proposed approach. “ASP” and “WEQ” represent “as-prepared” and “water-equilibrated”, respectively. The corresponding counter-ions are not shown in the schemes. (**b**) Chemical structures of the monomers and cellulose macromolecule. (**c**–**h**) Results of mechanical tests demonstrate dramatically enhanced mechanical properties: (**c**) tensile stress-strain curves and (**d**) corresponding Young’s modulus (*E*) and tensile fracture strength (*σ*_b_), (**e**) tearing force-extension curves and (**f**) corresponding tearing energy, (**g**) adhesive force-extension curves based on lap-shear tests and (**h**) corresponding adhesive strength. *ϕ*_CF_ = 6.0 wt%.

In this work, we develop a series of strong, tough, and adhesive composite hydrogels from polyampholyte (PA) gel reinforced by nonwoven cellulose-based fiber fabric (CF) sheets via a simple composite strategy (Figure 1a). Due to the fabrication method, the fibers in the pre-fabricated CF sheet could be aligned mainly in the plane direction (Figure S1). A relatively tough, sticky PA gel [i.e., P(NaSS-*co*-MPTC) gel, copolymerized from sodium *p*-styrenesulfonate (NaSS) and 3-(methacryloylamino)propyl-trimethylammonium chloride (MPTC)] is chosen as the matrix (Figure 1b). After integration with the PA gel matrix, CF could form a good interface with the hydrogel network through possible ionic and hydrogen bonds, providing the high load-bearing capability for PA/CF composite gels in the parallel direction. Large-aspect-ratio fibers could also effectively transfer and disperse stress widely in the composite gels, giving a large process zone to highly dissipate energy. Meanwhile, the composite gels are relatively soft (similar to the soft matrix) in the thickness direction, which could provide a large real contact area to realize high adhesion capacity. A systematic study on the effect of CF content on the mechanical and adhesion properties is carried out. The optimized composite gel exhibits 35.2 MPa of Young’s modulus, 4.3 MPa of tensile fracture strength, and 8.1 kJ m^−2^ of tearing energy, which is 22.1, 2.3, and 1.8 times those of the gel matrix, respectively. The optimized sample also shows not only high self-adhesion (943 kPa of adhesive strength and 1.4 kJ m^−2^ of adhesive energy, which is 6.0 and 4.2 times those of the gel matrix, respectively) but also good adhesion to diverse solid substrates. This study provides a simple but effective strategy to fabricate strong, tough, and adhesive hydrogel materials.

## 2. Materials and Methods

### 2.1. Materials

Sodium *p*-styrenesulfonate (NaSS, 90 wt%) was purchased from Macklin Biochemical Co., Ltd (Shanghai, China). 3-(methacryloylamino)propyl-trimethylammonium chloride (MPTC, 50 wt%) was purchased from J&K Chemical Ltd. *N*,*N*′-methylene-bis-acrylamide (MBAA) and *α*-ketoglutaric acid (*α*-keto) were purchased from Sinopharm Chemical Reagent Co., Ltd (Shanghai, China). Short cellulose-based fibers were laboratory-made from coniferous wood. The chemical structures of the monomers and cellulose molecule are shown in Figure 1b. All reagents are of analytical grade and were used as received. Deionized water (DI, 18.3 MΩ) was used in all the experiments.

### 2.2. Fabrication of CF Sheet

In order to achieve PA/CF composite hydrogels, a nonwoven cellulose-based fiber fabric (CF) sheet should be fabricated before integrating it with a pre-gel solution (Figure S2). First, 5.0 g of dry cellulose-based fibers was dissolved in 1000 mL DI water by magnetic stirring for 12 h to allow for a uniform dispersion, followed by a filtration treatment under a vacuum condition until a wet CF sheet was formed. Then, the wet CF sheet was dried for 2 h in an oven at 65 °C until a constant weight, achieving a dry CF sheet. To investigate CF content on mechanical and adhesive properties of PA/CF composite gels, 50, 100, 200, and 300 mL of the above CF solution were used for the vacuum-filtration and thermal-dehydration treatments to form CF sheets with different weights, corresponding to 1.5, 3.0, 6.0, and 9.0 wt% for PA/CF composite gels, respectively, in the next section.

### 2.3. Fabrication of PA/CF Composite Hydrogels

PA/CF composite hydrogels were fabricated via an in-situ free radical solution polymerization. Firstly, the nearly two-dimensional CF sheet (randomly oriented in the plane direction, as shown in Figure S1) was embedded into the reaction cell consisting of a pair of glass plates divided by a silicone spacer. The pre-gel solution containing anionic monomer (NaSS), cationic monomer (MPTC), crosslinker (MBAA), and photoinitiator (*α*-keto) was prepared according to the literature [12,37,38]. The total ionic monomer concentration (*C*_m_) and the molar fraction of anionic monomer were 2.2 mol L^−1^ and 0.49, respectively. The molar fractions of both crosslinker and initiator were 0.10 mol%, relative to *C*_m_. After that, the initial solution was injected into the above reaction cell, which was then kept in an oven at 65 °C for 30 min to guarantee sufficient diffusion of pre-gel solution into the cellulose-based fiber. Then, the reaction cell was irradiated by an ultraviolet lamp (365 nm, 4 W cm^−2^) for 8 h at ambient temperature to complete the polymerization. The fabrication process is illustrated in Figure S2. After polymerization, the as-prepared composite gels were obtained and were then immersed in adequate DI water for at least one week to remove the residual chemicals until a deswelling equilibrium was achieved. During this process, mobile counter ions were dialyzed gradually, and tremendous ionic bonds were formed between oppositely charged groups on the polymer chains; meanwhile, possible ionic and hydrogen bonds were also formed between the polymer chains and CF. As aforementioned, a series of PA/CF composite gels were fabricated by varying weight content (*ϕ* wt% *=* 1.5−9.0 wt%) of CF, and the samples were labeled as PA/CF-*ϕ*.

### 2.4. Tensile Tests

Tensile tests of the samples were carried out on a tensile tester (E43.104, MTS) with a 250 N load cell (standard: JIS-K6251-7). Before the tests, the samples were cut into a rectangle shape [length *l* (35 mm) × width *w* (10 mm) × thickness *t* (≈1 mm)], as illustrated in Figure S3. The sample width (*w* = 10 mm) for the tests was determined based on width-dependent tensile data, as presented in Figure S4 and Table S1. The tests were performed at room temperature with a velocity of 100 mm min^−1^. In order to prevent hydrogel dehydration, a humidifier was used during the tests. Young’s modulus, *E*, was calculated from the initial slope of the stress−strain curves at the stain within 10%. The work of tension of the samples during the tests, *W*_b_, was calculated by integrating the area under the stress-strain curves as follows:

(1) $$W_{b}=\int_{0}^{\varepsilon_{b}}\sigma d\varepsilon$$

where *σ* and *ε* are the stress and strain, respectively, and $\varepsilon_{b}$ is the strain at break of the samples. Each sample was tested at least three times, the average value was calculated, and the standard derivation was obtained as the error bar.

### 2.5. Tearing Tests

Trouser tearing tests of hydrogel samples were performed at room temperature to characterize the tearing energy, *T*. The samples were cut into a rectangular shape [*l* (40 mm) × *w* (20 mm) × *t* (≈1 mm)] with a 20 mm notch in the middle (standard: JIS-K6252 1/2), as illustrated in Figure S3. The two arms of the samples were clamped, and the upper arm was pulled at a velocity of 100 mm min^−1^ until the crack advanced through the entire sample, while the tensile force was recorded. The tearing energy, *T*, was calculated as follows:

(2) $$T=\frac{{2F}_{\mathrm{ave}}}{t}$$

where *F*_ave_ and *t* are the average tearing force to advance the crack and the sample thickness, respectively. Each sample was tested at least three times, the average value was calculated, and the standard derivation was obtained as the error bar.

### 2.6. Lap-Shear Tests

The adhesive strength of the hydrogel samples to diverse substrates (including hydrogels themselves) was characterized by lap-shear tests (standard: ASTM F2255) at ambient temperature. The samples were cut into a rectangular shape [*l* (70 mm) × *w* (20 mm) × *t* (≈1 mm)], and the overlapping length, *b*, between the hydrogels and substrates was kept constant at 15 mm, as illustrated in Figure S5. For self-lap-shear tests, the partially overlapped hydrogels were kept in an oven at 65 °C and were pressed by a 500 g weight on the surface for 1.5 h in order to guarantee sufficient true contact area. After that, the samples were soaked again in DI water for 2 h to avoid the dehydration influence in the last step. Thin polyester film was used as a flexible, inextensible backing for the hydrogel samples. A humidifier was used to supply a humid environment to minimize water evaporation of the samples during the tests. Each end of the hydrogel and the substrate was clamped with a tensile tester, and the shear velocity was 100 mm min^−1^. Adhesive strength, $\tau_{s}$, was calculated as follows:

(3) $$\tau_{s}=\frac{F_{\max}}{w\;\times \;b}$$

where *F*_max_ is the maximum force on the force-extension (*F*-Δ) curves. Each sample was tested at least three times, the average value was calculated, and the standard derivation was obtained as the error bar.

### 2.7. 90-Degree Peeling Tests

Interface toughness between the hydrogel samples was characterized by 90-degree peeling tests (standard: ASTM D2861) at ambient temperature. The samples were cut into a rectangular shape [*l* (70 mm) × *w* (20 mm) × *t* (≈1 mm)], and the overlapping length, *b*, between the hydrogels and substrates was kept constant at 15 mm, as illustrated in Figure S5. Before the tests, the partially overlapped hydrogels were kept in an oven at 65 °C and were pressed by a 500 g weight on the surface for 1.5 h in order to guarantee sufficient true contact area. After that, the samples were soaked again in DI water for 2 h to avoid the dehydration influence in the last step. Thin polyester film was used as a flexible, inextensible backing for the hydrogel samples. A humidifier was used to supply a humid environment to minimize water evaporation of the samples during the tests. The initial-crack end of the samples was clamped with a tensile tester, and the peeling velocity was 100 mm min^−1^. Photographs of the tests are shown in Figure S6. Interface toughness, *Γ*, was calculated as follows:

(4) $$\Gamma =\frac{F_{\mathrm{ave}}}{w}$$

where *F*_ave_ is the average plateau force in the steady-state region on the force-extension (*F*-Δ) curves, and *w* is the sample width. Each sample was tested at least three times, the average value was calculated, and the standard derivation was obtained as the error bar.

### 2.8. Water Content of Hydrogels

Water content (*ω*_w_) of composite hydrogels was tested using an electronic moisture meter (MOC-120H, Shimazu). The mode was set as automatic stop, and the temperature was set as 120 °C. *ω*_w_ was calculated as follows:

(5) $$\omega_{w}=\frac{m_{0\;}{-\;m}_{1\;}}{m_{0}}\times 100\%$$

where *m*_0_ and *m*_1_ are the weights of wet and dehydrated hydrogel samples, respectively. Each sample was tested at least three times, the average value was calculated, and the standard derivation was obtained as the error bar.

Other experimental details are presented in the Supporting Information.

## 3. Results and Discussion

### 3.1. Fabrication and Characterizations of Hydrogels

To achieve strong, tough, and adhesive composite hydrogels, we design to choose a relatively tough, sticky polyampholyte (PA) hydrogel as the matrix and nonwoven cellulose-based natural fiber fabric (CF) sheets as the reinforcement filler (Figure 1a). In detail, PA hydrogel consists of a P(NaSS-*co*-MPTC) network, which is copolymerized from sodium *p*-styrenesulfonate (NaSS) and 3-(methacryloylamino)propyl-trimethylammonium chloride (MPTC) with a small amount of chemical crosslinker (Figure 1a-i,ii) [12,37,38]. The dynamic ionic network (by an electronic attraction between oppositely charged groups on polymer chains) of the PA gel makes them mechanically tough and self-adjustably adhesive to diverse solid substrates [12,39]. Due to the fabrication method, the fibers in pre-fabricated CF sheets could be aligned mainly in the plane direction. Macroscopic and microscopic photographs of the CF sheet are given in Figure S2. In this design, we hypothesize that CF could interact effectively with the hydrogel network through possible ionic and hydrogen bonds among functional groups of the both components. In this case, large-aspect-ratio fibers in CF could transfer and disperse stress extensively in the composite gels, enabling high load-bearing and energy-dissipative capabilities. In addition, the relatively soft, sticky hydrogel matrix provides sufficient real contact area in the thickness direction of the sheet-shaped samples, achieving good adhesion.

The design strategy and fabrication process of the composite hydrogels are given in Figure 1a and Figure S2, respectively. After polymerization, as-prepared PA/CF (ASP-PA/CF) composite gels were obtained, followed by a dialysis process in water for around one week to reach an equilibrium, achieving water-equilibrated PA/CF (WEQ-PA/CF) composite gels. Photographs of PA/CF composite gels are presented in Figure S7. During this process, mobile counter ions in the PA network were dialyzed out, and tremendous ionic bonds were formed between oppositely charged groups on polymer chains, enabling a significant mechanical increase in the hydrogel matrix [12]. Furthermore, possible ionic and hydrogen bonds were also formed between the polymer chains and CF, enhancing the interfacial bonding, which should be beneficial for the mechanical properties of the composite gels. For simplicity, PA/CF was used instead of WEQ-PA/CF for the following discussion.

To validate our idea, we carried out a tensile test, tearing test, and lap-shear test on a representative PA/CF-6 composite hydrogel sample. The corresponding curves and detailed data are shown in Figure 1c–h and Table S2. A nonwoven CF sheet is also relatively weak because all single fibers just contact each other randomly, and the interaction is weak (Figure 1c,d). It is seen that the mechanical and adhesion properties of the PA/CF-6 composite gel are remarkably higher than these of the neat PA gel. In detail, PA/CF-6 composite gel exhibits 29.3 MPa of Young’s modulus and 4.3 MPa of tensile fracture strength, which is 18.3 and 2.2 times those of the PA gel matrix, respectively (Figure 1c,d). Meanwhile, PA/CF-6 composite gel also possesses improved tearing energy (*T* = 6.8 kJ m^−2^), which is 1.5 times that of the PA gel matrix (Figure 1e,f). The result confirms that our proposed strategy is effective for achieving composite hydrogels with high load-bearing and fracture-resistant capabilities. The enhanced tensile properties are mainly because the high-strength fibers could transfer and disperse stress in the composite gel, weakening the stress concentration. The improved tearing energy should be attributed to the good interfacial interaction between the gel matrix and the fibers as well as extensive stress transfer, which could significantly increase the process zone of the composite gel to highly dissipate energy widely when resisting a crack.

In addition to the mechanical properties, PA/CF-6 composite gel shows markedly improved adhesion strength ($\tau_{s}$ = 943 kPa), which is 6.0 times that of the PA gel matrix (Figure 1g,h). This improvement is mainly attributed to two possible reasons: (i) In the composite gel, a relatively soft, sticky gel matrix provided a large real contact area between the gels, while relatively strong fibers allowed the sample to bear much higher loads. (ii) The strong fibers in the bulk could also effectively transfer and disperse the interfacial stress to a larger area compared with the neat PA gel, enabling significantly high adhesion performance. After the test, the apparently larger deformation of the sample surface could be observed (Figure S8), evidencing the widespread stress transfer, although the failure of both neat and composite gels occurred mainly at the interfaces. These results well support our design strategy for the fabrication of strong, tough, and adhesive hydrogels.

**Figure 2:**
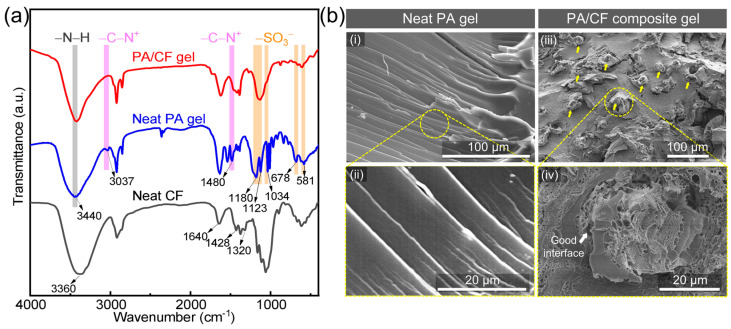
Structural characterizations of neat PA hydrogel and PA/CF composite hydrogel. *ϕ*_CF_ = 6.0 wt%. (**a**) FTIR spectra of the samples. Yellow, pink, and gray bars indicate the peaks of −SO_3_^−^, −(CH_3_)_3_N^+^, and −NH− groups, respectively. (**b**) SEM images of the fractured samples. Yellow arrows in (**iii**) indicate the existence of fractured fibers in the hydrogel matrix.

In order to understand the mechanical enhancement, the chemical structures of the neat and composite gels were also investigated by FTIR spectra (Figure 2a). On the spectrum of neat CF, the characteristic peaks at 3360 and 1320 cm^−1^ are related to −OH groups, and the peaks at 1640 and 1428 cm^−1^ are related to −COO^−^ groups. These characteristic peaks of −COO^−^ groups should be related to the existing hemicellulose, despite its limited amount in CF. On the spectrum of neat PA gel, the characteristic peaks at 1180, 1123, 1034, 678, and 581 cm^−1^ are related to −SO_3_^–^ groups, the peaks at 3037 and 1480 cm^−1^ are related to −(CH_3_)_3_N^+^ groups, and the peak at 3440 cm^−1^ are related to −NH− groups. On the spectrum of PA/CF composite gel, some characteristic peaks of these functional groups change in intensity or position, indicating the existence of some physical interactions between the two individual components. In detail, in PA/CF composite gel the characteristic peaks of −SO_3_^–^ groups at 1034, 678, and 581 cm^−1^ become weaker compared to neat PA gel; their peaks at 1180 and 1123 cm^−1^ become a single broad peak. The peak of −SO_3_^–^ groups at 581 cm^−1^ blueshifts to 607 cm^−1^ in PA/CF composite gel. Meanwhile, in PA/CF composite gel the peaks of −COO^−^ groups at 1640 and 1428 cm^−1^ redshift compared to neat CF. This result tells that some ionic bonds were probably formed between −(CH_3_)_3_N^+^ groups in the PA network and −COO^−^ groups in CF instead of some −(CH_3_)_3_N^+^•••^−^SO_3_− bonds. In addition, in PA/CF composite gel, the peak of −NH− groups at 3440 cm^−1^ becomes stronger and redshifts to 3419 cm^−1^, suggesting that hydrogen bonds were probably formed between −NH− groups in PA network and −OH or −COO^−^ groups in CF. These possible ionic and hydrogen bonds existing between the hydrogel matrix and CF could provide good interfacial interactions, enabling significant mechanical improvements.

Fracture surfaces of the hydrogel samples were further observed to understand the fracture behavior, and the corresponding SEM images are presented in Figure 2b. The neat PA gel shows a wrinkle-like fracture surface, demonstrating its relatively tough characteristic (Figure 2b-i,ii). Only the cross-sectional area of many fibers can be observed on the fracture surface of PA/CF composite gel, verifying the nonwoven fibers are mainly aligned in the plane direction (Figure 2b-iii). The magnified SEM image shows that a single fiber is surrounded by the hydrogel matrix compactly, and some hydrogel network seems to interpenetrate and interlock with the fiber (Figure 2b-iv). This observation directly confirms the good interfacial interactions between the hydrogel matrix and CF, in good agreement with the FTIR result described above. Based on the fracture surface of the composite gel, we can predict its fracture behavior mainly includes: (i) large deformation of the relatively tough gel matrix, (ii) fracture of the matrix, and (iii) fracture of the fibers. Such fracture behavior makes the PA/CF composite gel strong and tough.

### 3.2. Tensile and Tearing Behaviors of Hydrogels

**Figure 3:**
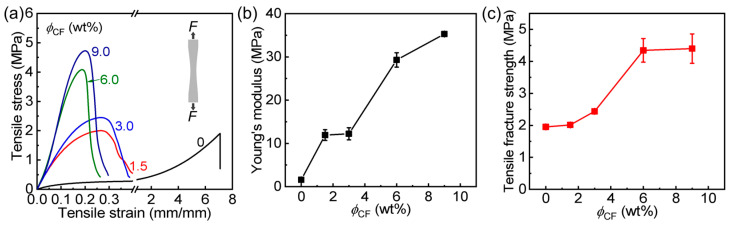
This is a figure. Schemes follow the same formatting. Tensile behavior of PA/CF composite hydrogels with different contents of cellulose-based fiber (*ϕ*_CF_). (**a**) Stress-strain curves of the samples. (**b**) Young’s modulus versus *ϕ*_CF_. (**c**) Tensile fracture strength versus *ϕ*_CF_.

Filler loading usually influences the mechanical reinforcement in composite gels [27]. Next, we investigate the effect of CF content (*ϕ*_CF_ = 1.5–9.0 wt%) on the mechanical properties of PA/CF composite gels. Tensile tests were first carried out, and the data are presented in Figure 3 and Table 1. The result shows that with increasing *ϕ*_CF_ (1.5–6.0 wt%), both Young’s modulus (*E*) and tensile fracture strength (*σ*_b_) of the samples first increase gradually, and after *ϕ*_CF_ > 6.0 wt%, the increase becomes relatively slow. When *ϕ*_CF_ is only 3.0 wt%, the sample was reinforced clearly: *E* = 12.2 MPa and *σ*_b_ = 2.4 MPa, which are 7.6 and 1.3 times these of neat PA gel, respectively. When *ϕ*_CF_ = 9.0 wt%, the optimal *E* and *σ*_b_ of the sample reach 35.3 MPa and 4.3 MPa, which are 22.1 and 2.3 times these of neat PA gel, respectively. This enhancement should be due to the fact that the strong fibers with good interfacial interactions with the matrix could effectively transfer and disperse the loads widely, which could reduce rapid stress concentration, enabling high load-bearing performance. However, excessive CF introduction might weaken the interfacial interaction, decreasing the enhancement efficiency of the composite gel. The introduction of CF probably shortened the effective stretch length of polymer chains in the hydrogel network, decreasing the tensile strain and work of extension of the composite gels.

**Figure 4:**
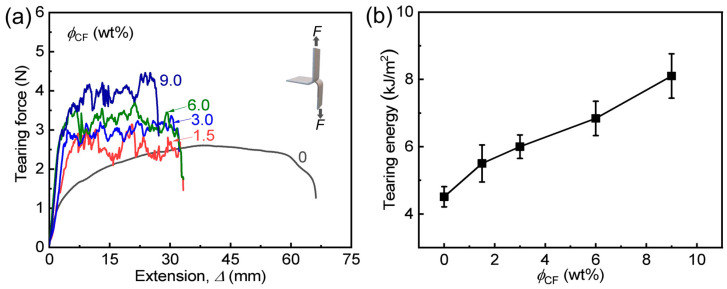
Tearing behavior of PA/CF composite hydrogels with different *ϕ*_CF_. (**a**) Tearing force (*F*)-extension (Δ) curves of the samples. (**b**) Tearing energy versus *ϕ*_CF_.

We further study the effect of CF content (*ϕ*_CF_ = 1.5–9.0 wt%) on the crack resistance (i.e., fracture toughness) of PA/CF composite gels by tearing tests, and the data are shown in Figure 4 and Table 1. Generally, the tensile strength and fracture toughness of a material are mutually exclusive. However, our composite gel system also provides improved crack resistance. With increasing *ϕ*_CF_ (1.5–9.0 wt%), tearing energy (*T*) increases gradually. When *ϕ*_CF_ is only 3.0 wt%, the sample could be toughened effectively, and *T* reaches 6.0 kJ m^−2^, which is 1.3 times of neat PA gel. When *ϕ*_CF_ increases further to 9.0 wt%, the maximized *T* highly reaches 8.1 kJ m^−2^, which is 1.8 times of neat PA gel. As aforementioned, introducing the strong, high-aspect-ratio fibers into the relatively tough, sticky PA gel could enlarge the process zone (i.e., energy-dissipative zone) remarkably, allowing it to highly dissipate energy broadly when resisting a crack. With increasing *ϕ*_CF_, the continuously increased *T* indicates that the energy dissipation of the hydrogel is still not saturated. However, when *ϕ*_CF_ > 9.0 wt%, the samples are difficult to fabricate successfully due to the high viscosity of the pre-solutions. It is worth noting that, despite mechanical enhancements, the composite gels still show increased water contents (*ω*_w_) compared to the neat PA gel (Figure S9). When *ϕ*_CF_ = 3.0 wt%, *ω*_w_ of the sample reaches 65.6 wt%, which is 1.2 times of the neat sample. This result should be attributed to the highly hydrophilic nature of cellulose-based CF. After *ϕ*_CF_ > 3.0 wt%, the slightly decreased *ω*_w_ should be mainly due to the increased interaction between CF and the matrix restricting the swelling of the hydrogel network, enabling further mechanical enhancements.

### 3.3. Adhesion Behaviors of Hydrogels

**Figure 5:**
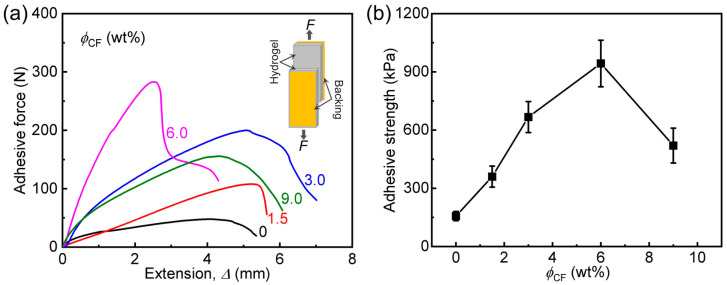
Lap-shear adhesion behavior of two pieces of PA/CF composite hydrogels with different *ϕ*_CF_. (**a**) Adhesive shear force (*F*)-extension (Δ) curves of the samples. (**b**) Adhesive strength versus *ϕ*_CF_.

**Figure 6:**
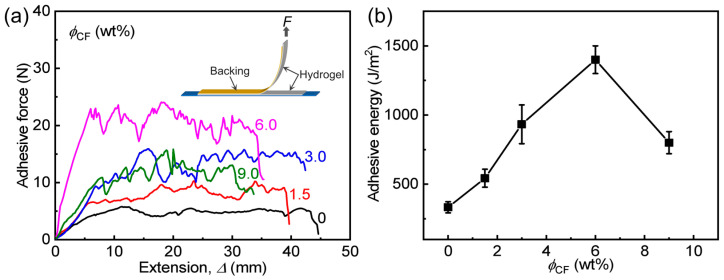
90-degree peeling adhesion behavior of two pieces of PA/CF composite hydrogels with different *ϕ*_CF_. (**a**) Peeling force (*F*)-extension (Δ) curves of the samples. (**b**) Adhesive energy versus *ϕ*_CF_.

**Figure 7:**
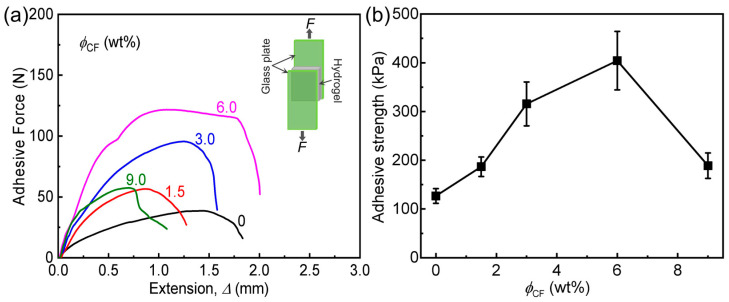
Adhesion behavior of PA/CF composite hydrogels with different *ϕ*_CF_ to a glass substrate. (**a**) Adhesive shear force (*F*)-extension (Δ) curves of the samples. (**b**) Adhesive strength versus *ϕ*_CF_.

**Figure 8:**
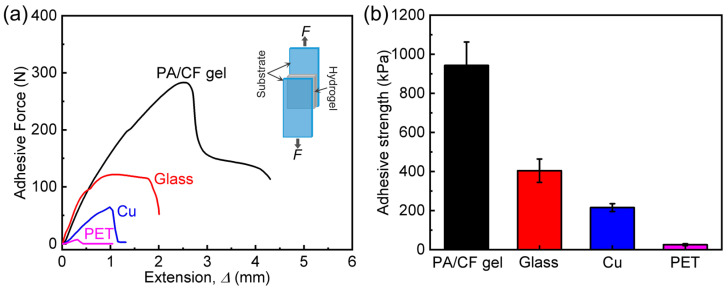
Lap-shear tests of PA/CF composite hydrogels on diverse substrates. These substrates include glass, metal (i.e., Cu), and plastic (i.e., PET). Self-lap-shear adhesion data of PA/CF gel is also included for comparison. *ϕ*_CF_ = 6.0 wt%. (**a**) Adhesive force (*F*)-extension (Δ) curves of the samples on different substrates. (**b**) Calculated adhesive strength of the samples on different substrates based on the tests.

As discussed above, filler loading could significantly influence the mechanical properties of the composite gels, which also probably results in different adhesion properties to diverse substrates. To clearly understand this point, here we systematically investigate the adhesion behaviors of the samples on different solid surfaces (including the gels themselves, glass, metal, and plastic) by lap-shear tests and 90-degree peeling tests. The detailed data are presented in Figure 5, Figure 6, Figure 7 and Figure 8, Table 2, and Table S3. We first evaluate the effect of CF content (*ϕ*_CF_) on adhesive strength ($\tau_{s,\;\mathrm{gel}}$) of two pieces of PA/CF composite gels by lap-shear tests. Adhesive force-extension (*F*-Δ) curves and the calculated $\tau_{s,\;\mathrm{gel}}$ of the samples are shown in Figure 5. From the *F*-Δ curves, we can find that each sample has a peak adhesive force before failure, in agreement with the literature [40,41,42]. All composite gels exhibit distinctly improved $\tau_{s,\;\mathrm{gel}}$, and *ϕ*_CF_ could clearly influence the lap-shear adhesion behavior. In detail, with increasing *ϕ*_CF_ (1.5–6.0 wt%), $\tau_{s,\;\mathrm{gel}}$ of the samples increases, and after *ϕ*_CF_ > 6.0 wt%, $\tau_{s,\;\mathrm{gel}}$ decreases but is still dramatically higher than that of neat PA gel. When *ϕ*_CF_ is only 1.5 wt%, the sample achieved a much higher $\tau_{s,\;\mathrm{gel}}$ (360 kPa), which is 2.3 times that of neat PA gel. When *ϕ*_CF_ = 6.0 wt%, the optimized sample possesses the highest $\tau_{s,\;\mathrm{gel}}$ (943 kPa), which is 6.0 times that of neat PA gel. The result demonstrates that our composite gels could adhere well to soft and wet surfaces.

We further evaluate the effect of *ϕ*_CF_ on adhesive energy (*Γ*) of two pieces of composite gels by 90-degree peeling tests. *F*-Δ curves and the calculated *Γ* of the samples are given in Figure 6. From the *F-*Δ curves, it is seen that each sample shows a sawtooth-like plateau adhesive force before failure, which is also usually observed in previous studies [34,35,43]. Similar to the lap-shear behavior (Figure 5), all composite gels have clearly higher *Γ* than the neat PA gel, and the samples with different *ϕ*_CF_ show different enhancements. In detail, with increasing *ϕ*_CF_ (1.5–6.0 wt%), *Γ* of the samples increases, and after *ϕ*_CF_ > 6.0 wt%, it decreases but is still higher than that of neat PA gel. When *ϕ*_CF_ is only 1.5 wt%, the sample achieved a much higher *Γ* (543 J m^−2^), which is 1.6 times that of neat PA gel. The optimized sample with *ϕ*_CF_ = 6.0 wt% has the highest *Γ* (1400 J m^−2^), which is 4.2 times that of neat PA gel.

As aforementioned, the enhanced adhesion properties based on these two kinds of tests should be attributed to: (i) relatively soft, sticky gel matrix provided good contact between the components while strong fibers could bear the relatively higher load. (ii) The strong fibers could also enlarge the energy-dissipative area in the bulk. According to a recent adhesion theory [34], the total interfacial toughness (*Γ*) of a hydrogel-solid bonding could be expressed as *Γ* = *Γ*_0_ + *Γ*_D_, where *Γ*_0_ is the intrinsic energy dissipation by the scission of a thin interfacial layer, and *Γ*_D_ is the energy dissipation from the bulk of the samples. Usually, *Γ*_D_ from the highly deformed bulk mainly contributes to the tough bonding. In our fabricated composite gels, varying *ϕ*_CF_ should mainly affect *Γ*_D_, enabling different enhancements described above. When keeping the same overlapping area (20 mm × 15 mm) of the samples, we can see that the maximum failure adhesive forces (*F*_f_) for both tests are quite different. When *ϕ*_CF_ = 6.0 wt%, *F*_f_ for the lap-shear test highly reaches >280 N, but *F*_f_ for the 90-degree peeling test is relatively low (around 20 N), giving a high parallel/vertical adhesion force ratio ($F_{f,∥}/F_{f,⊥}\;>\;14$). The high ratio means that the composite gels could bear a very high load in the parallel direction of the sample sheet, but it is very easy to release in the thickness direction. Such characteristic is very similar to the foot pads of geckos, exhibiting a good potential as a gecko-like soft and wet adhesive.

Adhesion of the composite gels to diverse solid substrates is important for their practical applications. Here we first study the adhesion behavior of the composite gels with different *ϕ*_CF_ to a glass substrate by lap-shear tests, and the data are shown in Figure 7. Similarly, adhesion strength ($\tau_{s,\;\mathrm{glass}}$) of the samples increases gradually with increasing *ϕ*_CF_ (1.5–6.0 wt%), and then decreases after *ϕ*_CF_ > 6.0 wt%. When *ϕ*_CF_ is only 1.5 wt%, $\tau_{s,\;\mathrm{glass}}$ of the sample is 187 kPa, which is 1.5 times that of neat PA gel. The sample with 6.0 wt% CF possesses the highest $\tau_{s,\;\mathrm{glass}}$ (404 kPa), which is 3.2 times that of neat PA gel. The result tells that the composite gels also show remarkably enhanced adhesion performance to glass substrate compared to the neat gel based design strategy. Finally, we further evaluate and compare the adhesion strength ($\tau_{s}$) of the composite gel (*ϕ*_CF_ = 6.0 wt%) to diverse substrates (including glass, Cu, and PET) by lap-shear tests, as shown in Figure 8 and Table S3. Clearly, the composite gels exhibit quite different $\tau_{s}$ to different substrates: composite gel > glass > Cu > PET. The difference in $\tau_{s}$ should be mainly attributed to the self-adjustable adhesion nature of the ionic PA network, which tends to adhere to the substrates with high polarity. $\tau_{s}$ of the composite gels to these polar substrates (i.e., composite gel, glass, and Cu) is relatively high (>200 kPa), which surpasses many existing hydrogel adhesives [41,42,44,45,46]. However, $\tau_{s}$ of the composite gels to the non-polar plastic (i.e., PET) is relatively limited because of the limited intrinsic interfacial energy (*Γ*_0_). All of the above results demonstrate that high strength, high fracture toughness, and good adhesion could be achieved simultaneously in PA/CF composite hydrogels.

## 4. Conclusions

In summary, we have developed a series of strong, tough, and adhesive composite hydrogels from relatively tough, sticky polyampholyte (PA) gel reinforced by cellulose-based nonwoven fiber fabric (CF) via a composite strategy. In PA/CF composite gels, strong fibers in CF could interact with the relatively tough matrix well through possible ionic and hydrogen bonds, enabling effective stress transfer and dispersion (high strength) as well as wide energy dissipation (high toughness). Meanwhile, the soft, sticky matrix could also provide a large contact area to realize good adhesion. The effect of CF content (*ϕ*_CF_, 1.5–9.0 wt%) on the mechanical and adhesion properties of composite gels was systematically studied. The results show that *ϕ*_CF_ could dramatically influence both the mechanical and adhesion properties. The optimized composite gel achieved 35.2 MPa of Young’s modulus, 4.3 MPa of tensile fracture strength, 8.1 kJ m^−2^ of tearing energy, 943 kPa of self-adhesive strength, and 1.4 kJ m^−2^ of self-adhesive energy, which is 22.1, 2.3, 1.8, 6.0, and 4.2 times those of neat PA gel, respectively. Additionally, the composite gels could also adhere well to diverse solid substrates (including glass, metal, and plastic). This work opens a simple yet effective pathway to achieve soft and wet materials with high strength, high toughness, and good adhesion.

## Figures and Tables

**Table 1:** Summary of mechanical properties and water content of PA and PA/CF hydrogels.

Sample code (*ϕ*) ^(a)^	*E* (MPa)	*σ*_b_ (MPa)	*W*_b_ (MJ m^−3^)	*T* (kJ m^−2^)	*ω*_w_ (wt%)
PA	1.6 ± 0.1	1.9 ± 0.1	5.9 ± 0.1	4.5 ± 0.3	53.5 ± 1.9
PA/CF-1.5	11.9 ± 1.2	2.0 ± 0.1	0.50 ± 0.05	5.5 ± 0.6	57.1 ± 0.1
PA/CF-3	12.2 ± 1.4	2.4 ± 0.1	0.70 ± 0.04	6.0 ± 0.4	65.6 ± 1.3
PA/CF-6	29.3 ± 1.7	4.3 ± 0.4	0.60 ± 0.15	6.8 ± 0.5	62.9 ± 1.0
PA/CF-9	35.3 ± 0.1	4.3 ± 0.5	0.80 ± 0.03	8.1 ± 0.7	60.6 ± 0.4

^(a)^*ϕ* in the code of PA/CF-*ϕ* represents weight percentage (wt%) of CF in the corresponding PA/CF composite hydrogels.

**Table 2:** A summary of the adhesion properties of PA/CF composite hydrogels with different CF contents (shown in Figure 5, Figure 6 and Figure 7).

Sample Code	$\tau_{s,\;\mathrm{gel}}$ (kPa) ^(a)^	*Γ* (J m^−2^)	$\tau_{s,\;\mathrm{glass}}$ (kPa) ^(b)^
PA	157 ± 24	333 ± 40	127 ± 15
PA/CF-1.5	360 ± 54	543 ± 65	187 ± 20
PA/CF-3	667 ± 80	933 ± 140	316 ± 45
PA/CF-6	943 ± 120	1400 ± 100	404 ± 60
PA/CF-9	520 ± 90	800 ± 80	189 ± 26

^(a)^ $\tau_{s,\;\mathrm{gel}}$ is the adhesive strength of two pieces of hydrogels. ^(b)^ $\tau_{s,\;\mathrm{glass}}$ is the adhesive strength of hydrogels on glass substrate.

## Data Availability

Not applicable.

## Supplementary Materials

The following supporting information can be downloaded at: https://www.mdpi.com/article/10.3390/polym14224984/s1, Figure S1: Macroscopic and microscopic images of cellulose-based fiber fabric (CF) sheet used in this work; Figure S2: Fabrication process of PA/CF composite hydrogels; Figure S3: Sample geometries and methods for tensile and tearing tests; Figure S4: Mechanical properties of PA/CF composite hydrogels with different sample widths; Figure S5: Sample geometries and methods for adhesion tests; Figure S6: Photographs of 90-degree peeling tests for PA hydrogel and PA/CF composite hydrogels; Figure S7: Macroscopic photographs of PA/CF composite hydrogels; Figure S8: Failure surface of two pieces of PA/CF composite hydrogels after the lap-shear test; Figure S9: Water content (*ω*_w_) versus *ϕ*_CF_ for PA/CF composite hydrogels; Table S1: Summary of tensile properties of PA/CF composite hydrogels with different sample widths shown in Figure S4; Table S2: Summary of tensile properties of representative hydrogel samples shown in Figure 1c–e; Table S3: Summary of adhesion strength of PA/CF-6 composite hydrogels to diverse substrates shown in Figure 8.
