# Supplementary material for: Strong, Tough, and Adhesive Polyampholyte/Natural Fiber Composite Hydrogels
Source: Polymers (Basel). 2022 Nov 17;14(22):4984. doi: 10.3390/polym14224984 (PMC9699137; doi:10.3390/polym14224984)
Supplement: Supplementary file 1 [file polymers-14-04984-s001.zip › polymers-2027769-supplementary.pdf]

Supplementary Material: Strong, Tough, and Adhesive Polyampholyte/ Natural Fiber Composite Hydrogels

Yongqi Yan, Longya Xiao, Qin Teng, Yuanyuan Jiang, Qin Deng, Xuefeng Li and Yiwan Huang

S1. Supplementary Methods

S1.1. Fourier Transform Infrared Spectroscopy (FTIR)

FTIR spectra of the hydrogel samples were recorded by using a Tensor 2 FTIR spectrometer (NeXus, USA). The measured specimens were prepared by the KBr-disk method, and the spectra in the wavenumber range of 4000–400 cm^{-1} were collected with a resolution of 4 cm^{-1}. All the experiments were carried out at ambient temperature.

S1.2. Field Emission Scanning Electron Microscopy (FESEM)

Fracture morphologies of the hydrogel samples were observed by a Hitachi SU8010 FESEM. In detail, the samples were frozen and fractured in liquid nitrogen and then freeze-dried for 24 hours. Afterwards, the fractured surfaces of the samples were gold-coated in a JUC-500 Magnetron Sputtering Device (JEOL, Tokyo, Japan), and then observed with an accelerating voltage of 5 kV.

S2. Supplementary Figures

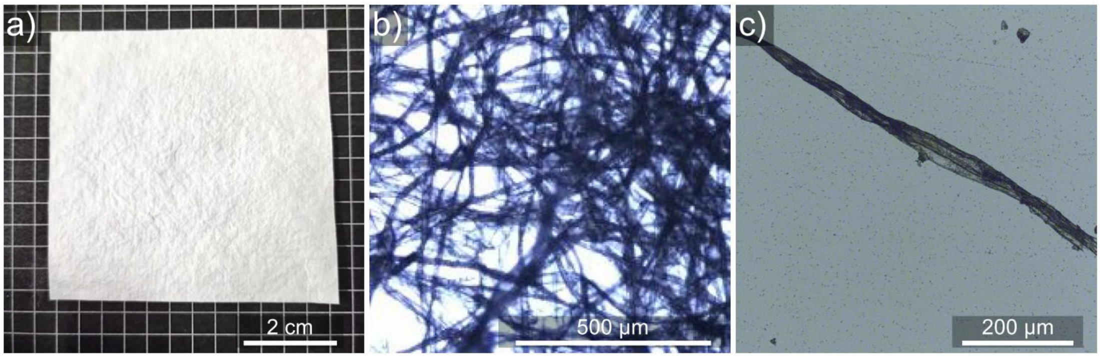

Figure S1. Macroscopic and microscopic images of cellulose-based fiber fabric (CF) sheet used in this work. (**a**) Macroscopic photograph of the dried CF sheet. (**b**,**c**) Optical microscopic photographs of the CF sheet (**b**) and a single fiber (**c**). Geometry information of the single fiber: 20–50 μm in diameter and 1.5–2 mm in length.

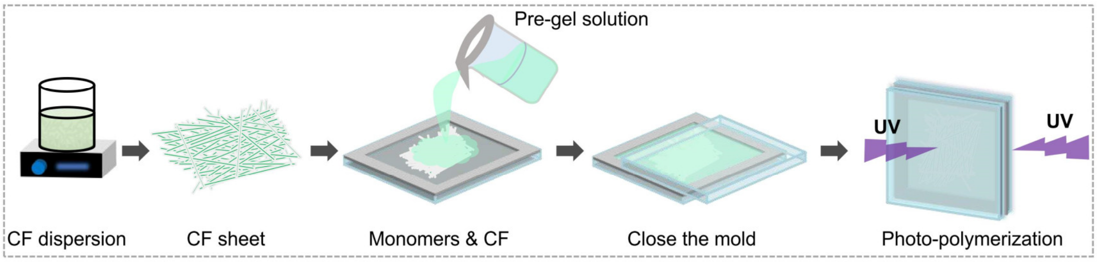

Figure S2. Fabrication process of PA/CF composite hydrogels.

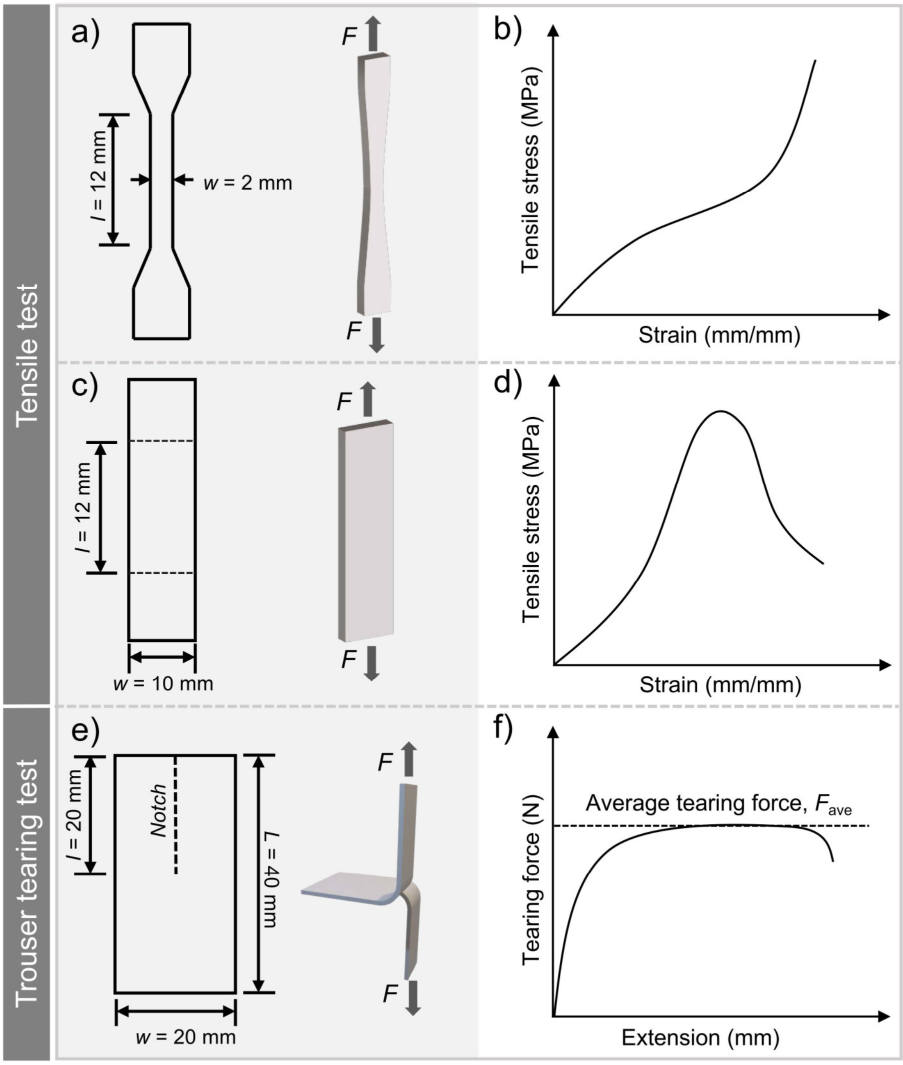

Figure S3. Sample geometries and methods for tensile and tearing tests. (**a,b**) Geometry and a representative stress-strain curve of PA gel in tensile tests. (**c,d**) Geometry and a representative stress-strain curve of PA/CF composite gels in tensile tests. (**e,f**) Geometry and a representative tearing force-extension curve of PA/CF composite gels in tearing tests. Average force (F_{ave}) obtained from the curve is used for calculation of tearing energy according to the literatures.

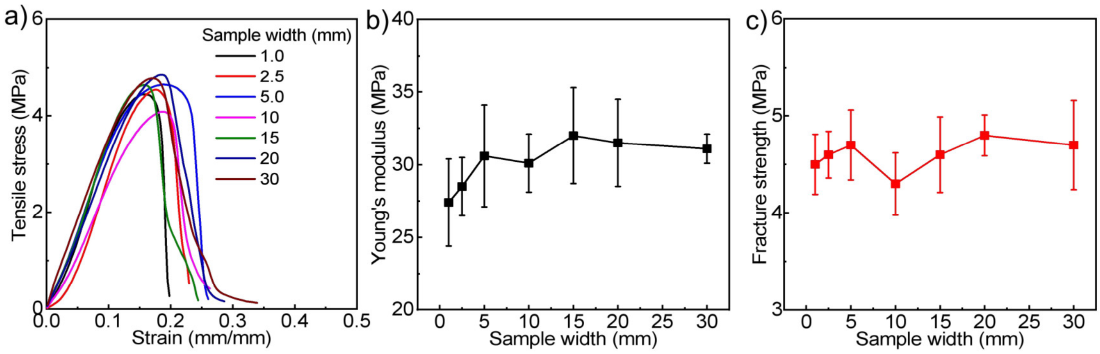

Figure S4. Mechanical properties of PA/CF composite hydrogels with different sample widths. ϕ_{CF} = 6.0 wt%. (**a**) Stress-strain curves. (**b**) Young's modulus. (**c**) Tensile fracture strength. Due to the geometrical effect, the composite gel could achieve relatively constant mechanical properties after sample width $w > 5$ mm.

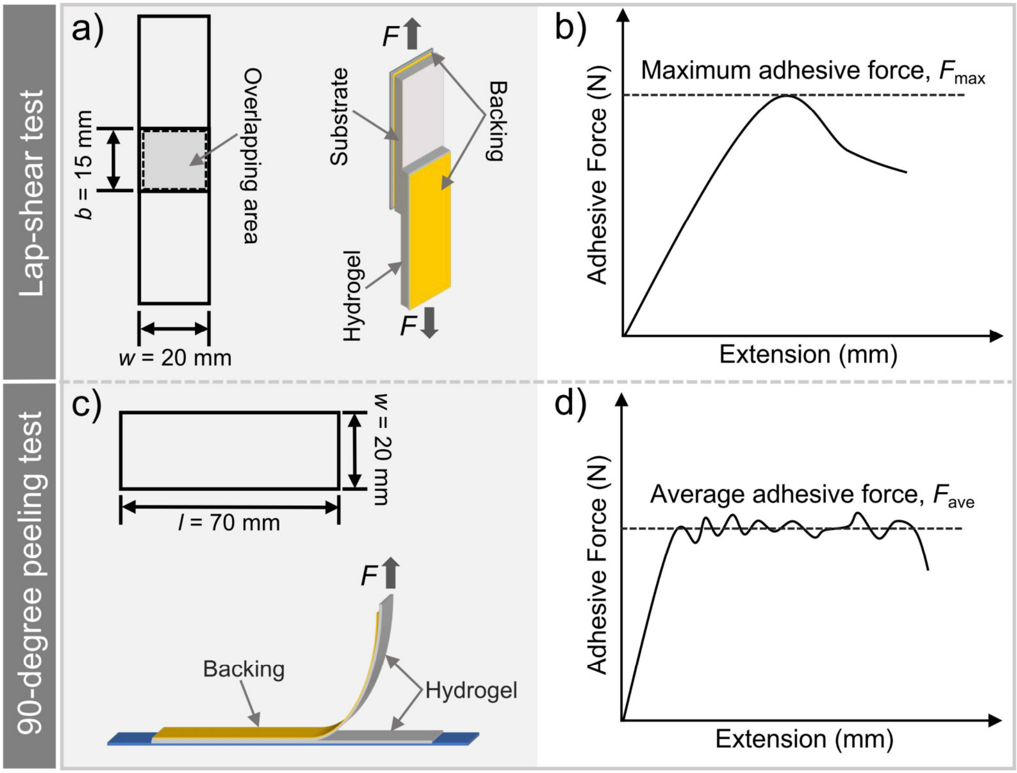

Figure S5. Sample geometries and methods for adhesion tests. (**a,b**) Geometry and a representative adhesive force-extension curve of PA/CF composite hydrogels in lap-shear tests. Maximum adhesive force (F_{max}) obtained from the curve is used for calculation of adhesive strength. (**c,d**) Geometry and a representative adhesive force-extension curve of PA/CF composite hydrogels in 90-degree peeling tests. Average adhesive force (F_{ave}) obtained from the curve is used for calculation of adhesive energy according to the literatures.

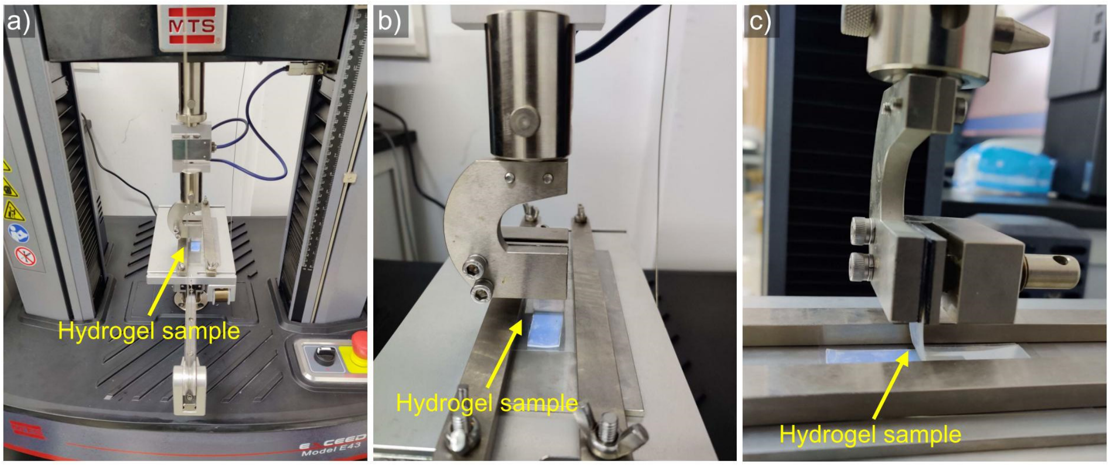

Figure S6. Photographs of 90-degree peeling tests for PA hydrogel and PA/CF composite hydrogels.

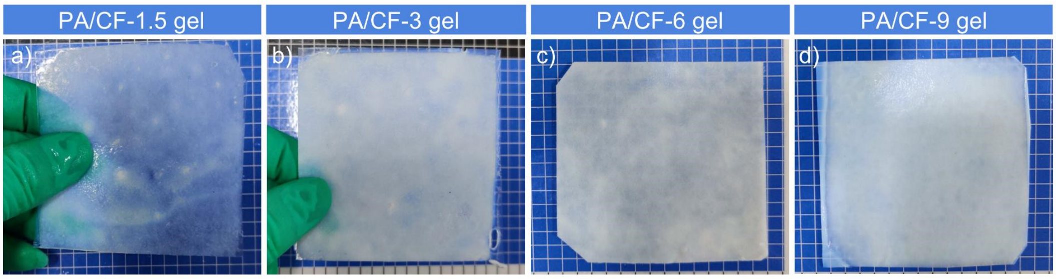

Figure S7. Macroscopic photographs of PA/CF composite hydrogels.

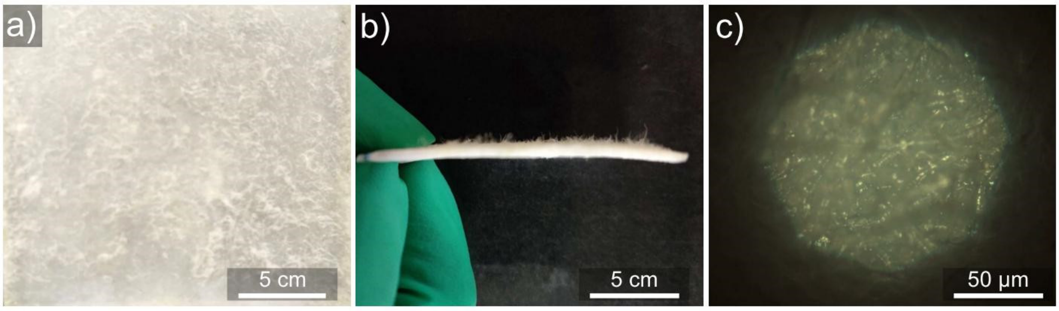

Figure S8. Failure surface of two pieces of PA/CF composite hydrogels after the lap-shear test. ϕ_{CF} = 6.0 wt%. (**a,b**) Macroscopic photographs in two directions. (**c**) Optical microscopic photograph.

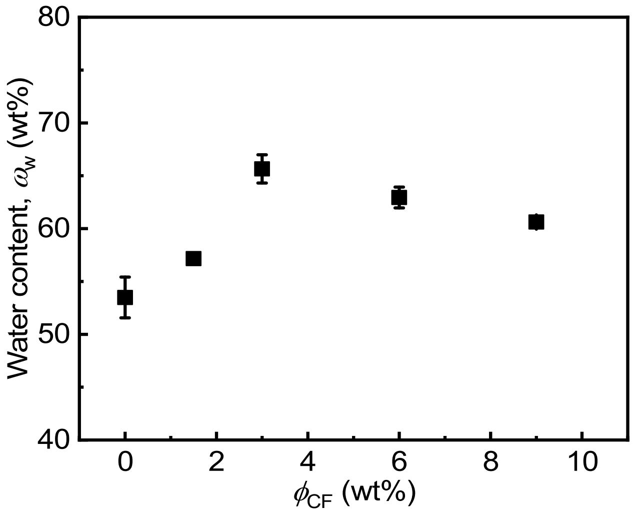

Figure S9. Water content (ω_w) versus ϕ_{CF} for PA/CF composite hydrogels.

S3. Supplementary Tables

Table S1. Summary of tensile properties of PA/CF composite hydrogels with different sample widths (shown in **Figure S4**).

Sample width (mm)	*E* (MPa)	σ_b (MPa)	W_b (MJ m^{-3})
1.0	27.4 ± 3.0	4.5 ± 0.3	0.53 ± 0.05
2.5	28.5 ± 2.0	4.6 ± 0.2	0.67 ± 0.06
5.0	30.6 ±3.5	4.7 ± 0.4	0.79 ± 0.07
10	30.1 ± 2.0	4.3 ± 0.3	0.58 ± 0.04
15	32.0 ± 3.3	4.6 ± 0.4	0.60 ± 0.03
20	31.5 ± 3.0	4.8 ± 0.2	0.73 ± 0.05
30	31.1 ± 1.0	4.7 ± 0.4	0.69 ± 0.06

Table S2. Summary of tensile properties of representative hydrogel samples (shown in **Figure 1c,d,e**).

**Sample code (ϕ)** [a]	**E (MPa)**	**σ_b (MPa)**	**W_b (MJ m^{-3})**
Neat PA gel	1.7 ± 0.3	2.0 ± 0.2	5.9 ± 0.4
PA/CF-ϕ gel	29.3 ± 1.7	4.3 ± 0.4	0.6 ± 0.1
Neat CF sheet	10.4 ± 0.9	0.3 ± 0.1	0.01 ± 0.01

[a] ϕ in the code of PA/CF-ϕ represents weight percentage of CF in the corresponding PA/CF composite hydrogel.

Table S3. Summary of adhesion strength of PA/CF-6 composite hydrogels to diverse substrates (shown in **Figure 8**).

**Substrates**	**PA/CF-6 hydrogel**	**Glass**	**Cu**	**PET**
τ_s (kPa)	940 ± 120	404 ± 60	215 ± 20	25 ± 5
